# Proof-of-Concept Automated Framework for Intraoperative Transesophageal Echocardiography: View Classification and Biventricular Function Assessment

**DOI:** 10.1053/j.jvca.2026.01.036

**Published:** 2026-01-28

**Authors:** Trevor Chan, Shir Goldfinger, Rachel Hannah Grasfield, Vikram Eswar, David Barreto, John G. Augoustides, Vivian Yan, Ronak M. Shah, Alison M. Pouch, Emily J. MacKay

**Affiliations:** * Department of Radiology, University of Pennsylvania, Philadelphia; † Department of Bioengineering, University of Pennsylvania, Philadelphia; ‡ School of Arts and Sciences, University of Pennsylvania; § School of Medicine, Des Moines University, Des Moines, IA; || Department of Anaesthesiology and Critical Care, Perelman School of Medicine at the University of Pennsylvania, Philadelphia; ¶ School of Medicine, Thomas Jefferson University, Philadelphia, PA; ** Penn’s Cardiovascular Outcomes, Quality and Evaluative Research Center (CAVOQER), University of Pennsylvania, Philadelphia

**Keywords:** deep learning, echocardiography, perioperative medicine, cardiac surgery

## Abstract

**Objective:**

To develop a proof-of-concept automated framework for integrated intraoperative transesophageal echocardiography (TEE) interpretation, encompassing view classification, left ventricular ejection fraction (LVEF) and right ventricular systolic function (RVSF) assessment, and tricuspid regurgitation (TR) grading.

**Design:**

This cross-sectional study used TEE video clips and reports from patients undergoing cardiac surgery from 2018 to 2023.

**Setting:**

Two hospitals within the University of Pennsylvania Healthcare System.

**Participants:**

Study participants were ≥ 18 years of age and undergoing any cardiac surgery in which an intraoperative TEE was performed. We used 6,900 intraoperative TEE studies from 6,016 unique patients.

**Interventions:**

Receipt of intraoperative TEE examination during cardiac surgery.

**Measurements and Main Results:**

The view classification model was trained on TEE clips from 1,432 studies, and the diagnostic model was trained on clips from 6,400 studies, for a total of more than 700,000 individual TEE clips for training. Performance was evaluated on 945 test clips from 8 studies for view classification and 500 studies for diagnostic prediction. No external testing was performed. The view classifier achieved 86% accuracy compared to the expert agreement top-1 weighted accuracy of 74%. The diagnostic model differentiated LVEF >55% versus ≤ 55% (area under the receiver operating characteristic curve [AUROC] = 0.86) and LVEF >30% versus ≤ 30% (AUROC = 0.95). Continuous LVEF prediction was assessed (Pearson r = 0.79, mean absolute error = 7.8). The model differentiated normal versus abnormal RVSF (AUROC = 0.82) and differentiated less-than-moderate versus moderate-or-greater right ventricular dysfunction (AUROC = 0.92). Additionally, the model differentiated no TR versus any TR (AUROC = 0.69) and differentiated less-than-moderate versus moderate-or-greater TR (AUROC = 0.79).

**Conclusions:**

This proof-of-concept automated approach showed encouraging results for view classification and quantification tasks, suggesting potential for further development toward intraoperative utility.

ECHOCARDIOGRAPHY IS THE MOST WIDELY used cardiac imaging modality and plays a pivotal role in the diagnosis, monitoring, and intraoperative management of the more than 21 million people living in the United States with cardiovascular disease.^1,2^ Despite its ubiquity, echocardiographic interpretation remains heavily dependent on operator expertise, introducing substantial variability into both image acquisition and diagnostic assessment.^3–5^ In response, recent years have seen rapid growth in deep learning-based approaches to automate echocardiography interpretation, with the goal of enhancing diagnostic consistency, reducing workload, and improving clinical efficiency.^6–9^

Progress in artificial intelligence (AI) applications for echocardiography has largely centered on transthoracic echocardiography (TTE), where deep learning models have demonstrated high performance in tasks such as view classification,^10–16^ chamber segmentation,^12,17^ ejection fraction (EF) estimation,^12,15,17^ and disease phenotyping.^12,15,18^ Notably, a recent study introduced PanEcho, an automated deep-learning system for echocardiogram analysis, and highlighted the scalability of multitask models trained on large TTE datasets. PanEcho achieved robust performance across 18 classification and 21 regression tasks while generalizing across institutions and acquisition settings.^15^ However, similar efforts in transesophageal echocardiography (TEE), particularly in the intraoperative cardiac surgical setting, remain relatively underexplored.^19^

Intraoperative TEE is an essential imaging modality used during cardiac surgeries, facilitating real-time clinical decision-making^20–22^ and contributing to improved surgical outcomes.^23–27^ Compared to TTE, TEE offers superior spatial resolution due to reduced acoustic impedance (i.e., better “windows”).^28,29^ A typical TEE exam also captures a broader array of views (standardized and nonstandardized) captured over a longer timeframe. Comprehensive intraoperative TEE exams performed during cardiac surgery may include hundreds of video clips acquired across presurgical and postsurgical interventions, pre– and post–cardiopulmonary bypass stages, and in the context of complex pathology (i.e., valvular disease, biventricular dysfunction, aortic aneurysms, and mechanical circulatory support). Additionally, the intraoperative environment introduces unique challenges such as imaging with prosthetic materials, postsurgical changes, and patient-specific anatomic factors (e.g., mitral annular calcification or aortic aneurysms), all of which can introduce echocardiographic artifacts that complicate image interpretation.^30^ Together, these factors make intraoperative TEE a compelling, yet technically demanding, candidate for AI-facilitated automated interpretation.

In this study, we aimed to develop a proof-of-concept, deep learning–based method for intraoperative TEE that accomplishes the tasks of view classification, diagnostic prediction of biventricular dysfunction (left ventricular [LV] EF [LVEF] and right ventricular [RV] systolic function [RVSF]), as well as classification of tricuspid regurgitation (TR) severity. Due to the critically important relationship between TR and RVSF, TR was included in this analysis. Our goal was to develop a foundational framework for automated intraoperative TEE interpretation that may inform future applications in real-time, AI-supported decision-making during cardiac surgery.

## Methods

### Representative Perioperative TEE Dataset With Annotations

Data used in this study consisted of more than 740,000 intraoperative TEE video clips drawn from 6,900 cardiac surgical cases performed at the Hospital of the University of Pennsylvania (HUP) and Pennsylvania Presbyterian Medical Center (PPMC) between December 3, 2018, and July 17, 2023. For the purposes of this study, each TEE examination acquired during a case was referred to as a study, and each study was associated with an intraoperative TEE report authored by one of several (>10) cardiac anesthesiologists as part of their routine clinical documentation. Each intraoperative TEE study routinely consisted of distinct preintervention and postintervention imaging exams, acquired before and after cardiopulmonary bypass. Correspondingly, the associated intraoperative TEE report contained separate sections documenting preintervention and postintervention assessments of LVEF, RVSF, and TR severity. The 6,900 studies corresponded to 6,016 unique patients.

The studies included a broad range of cardiac surgical procedures (Table 1). Common procedures included isolated coronary artery bypass grafting; valve surgeries such as aortic, mitral, and tricuspid valve replacement or repair; and combined valve and bypass procedures. Cases such as heart and lung transplants were also represented. Additionally, the dataset included patients undergoing ventricular assist device implantation or explantation, as well as extracorporeal membrane oxygenation support. This diverse set of procedures allowed for analysis of intraoperative TEE across a wide range of surgical contexts.

From this dataset, 10,741 clips drawn from 1,432 studies were used to train the view classification model, and 945 clips from 8 randomly selected studies were reserved for model testing. For the biventricular function and TR severity diagnostic model, 6,400 studies (with 6,400 corresponding reports) were used for model training, and 500 randomly selected studies (with 500 corresponding reports) were reserved for model testing. This study was reviewed and approved as exempt by the university institutional review board.

### Data Annotation

**Fig 1.**
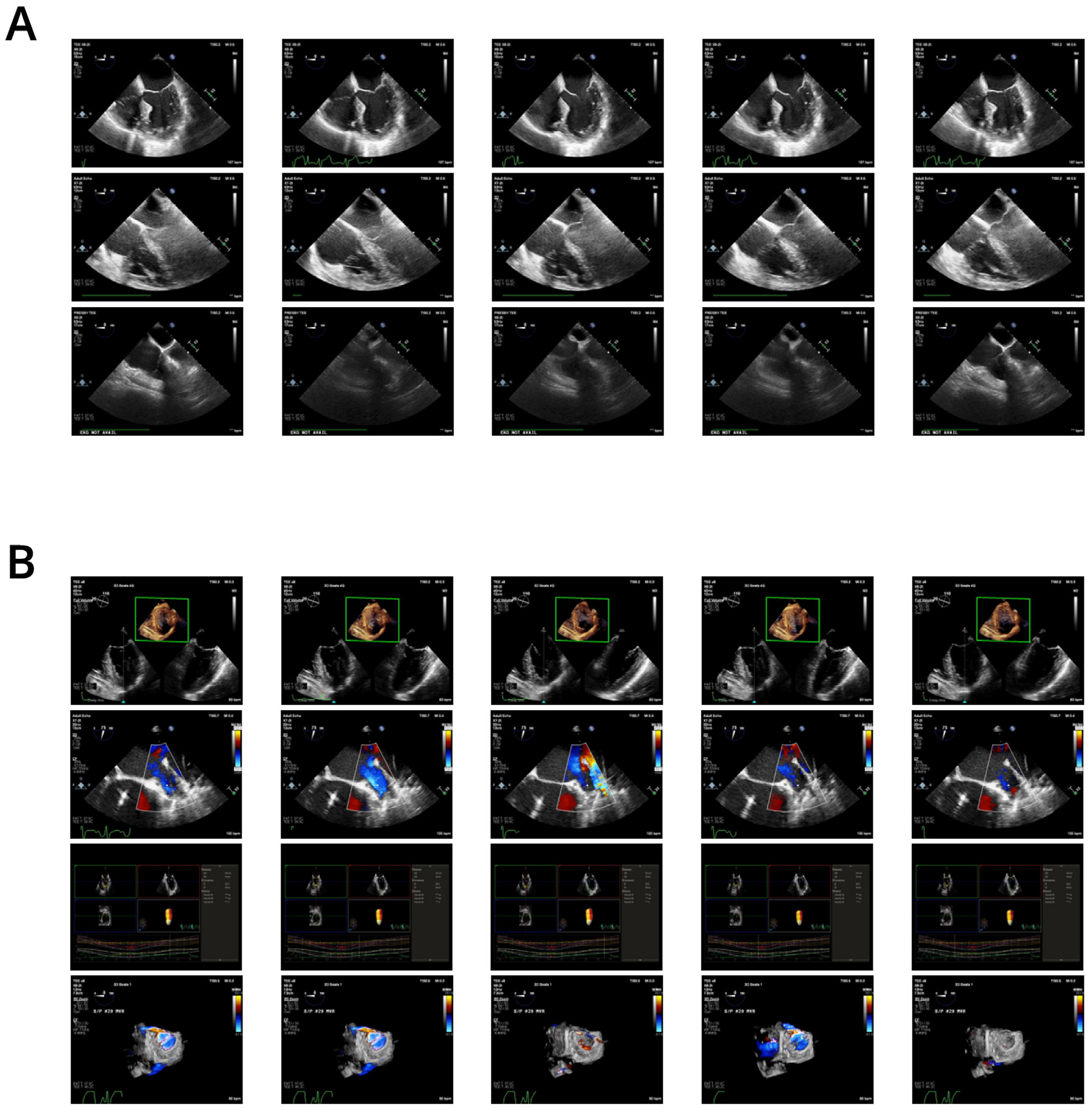
Image quality variability and nonclassifiable TEE views. Image quality varies dramatically within and across imaging studies. (A) Three midesophageal 4-chamber views depicting (*top*) ideal image quality, characterized by high contrast between chambers and heart walls, sharp mitral and tricuspid valves, and both ventricles fully within the field of view; (*middle*) typical image quality, in which most structures are visible but with lower contrast or some borders obscured by shadowing; and (*bottom*) poor image quality, in which the majority of structures are obscured by artifacts or outside the field of view. (B) A large number of clips were not classifiable within the 26 standard views. These included multipanel images, views acquired from unlabeled probe positions, motion studies, and 3-dimensional renderings.

Sources differ on the exact number of standard TEE views, but a general consensus identifies two-dimensional (^20–30^) TEE views commonly acquired during a comprehensive imaging examination.^22,31^ We found that a large number of the clips in our dataset do not cleanly categorize into these common views. Significantly degraded images were also common throughout the dataset (Fig 1, A), and clinicians often made alterations to transducer orientation and field of view to focus on regions of interest or avoid sources of image artifacts, complicating labeling. In this study, the list of views was narrowed down to the 26 views of interest with the highest frequency in the dataset (Table 2). All other views were assigned the miscellaneous label “other.” This study additionally assigned all M-mode images, two-dimensional renderings of three-dimensional images, and x-plane images in the dataset to the “other” category (Fig 1, B). View class annotation of all training, validation, and testing data was performed manually and reviewed by a board-certified cardiac anesthesiologist. A subset of the data was additionally annotated by a second cardiac anesthesiologist to assess interobserver agreement.

To accelerate the data annotation process for view classification, we implemented a multistage feedback labeling process. At each stage/cycle, the current classification model generates predicted labels for a set of unlabeled clips. Annotators then reviewed these predictions, marking each as either correct or incorrect. All clips marked correct were added to the training set together with their predicted label; clips marked incorrect were excluded from both the training set and further relabeling. This approach avoided the need for annotators to assign labels from scratch, thereby reducing labeling time and effort. In total, one initial fully supervised training stage (cycle 0: 5,890 clips from 42 studies) and 3 cycles of feedback labeling (cycle 1: 1,749 clips from 496 studies; cycle 2: 2,367 clips from 656 studies; cycle 3: 735 clips from 238 studies) were performed. After each cycle, the model was retrained on the augmented dataset, leading to continuously improved model accuracy. Together, these cycles represent the 10,741 clips drawn from 1,432 studies used for model training.

Training annotations for biventricular function and TR severity were obtained using a large language model (LLM) ensemble method outlined in previous work by our group.^32^ Each study was associated with a linked TEE report, and the ensemble method allowed for the extraction of structured preintervention and postintervention echocardiographic parameters from the unstructured report text. We used 6,400 intraoperative TEE reports for model training. Echocardiographic parameters were extracted by the LLM method according to current American Society of Echocardiography, American College of Cardiology, and American Heart Association guidelines.^33^ LVEF was extracted as a numerical percentage, while RVSF and TR were extracted as categorical values. RVSF was extracted as normal, borderline, mildly, mild-to-moderately, moderately, moderate-to-severely, or severely decreased function, and TR severity as none, trivial/trace, mild, mild-to-moderate, moderate, moderate-to-severe, or severe.^33^ These categorical values reflected the language and grading scales used in the TEE reports.

### Model Architecture and Training

**Fig 2.**
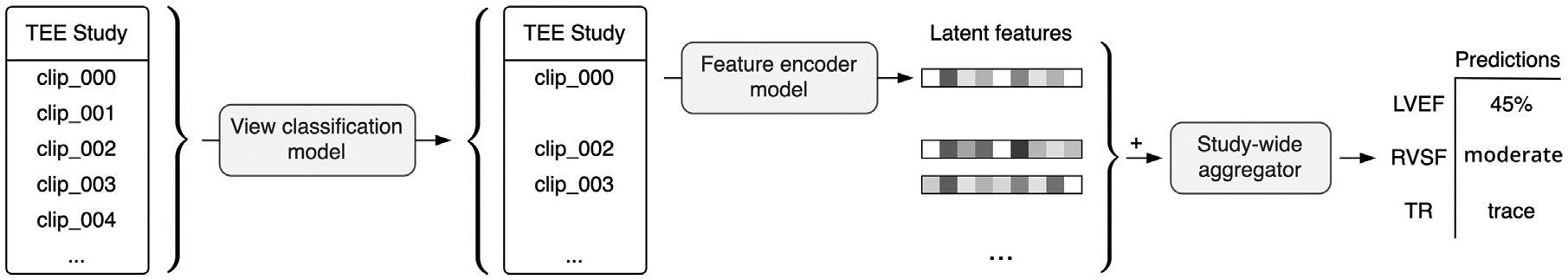
Workflow for automated intraoperative TEE analysis. Automated analysis of an intraoperative TEE study involves a multistep process in which clips are first classified by view and filtered to obtain useful views. Next, features are extracted on a per-clip basis. Finally, the aggregated features are passed to a neural network to give a diagnostic prediction.

Our approach to complete study analysis divided the task of image interpretation into 3 steps: an initial view classification step and 2 steps for diagnostic prediction (Fig 2). The first step used a convolutional neural network to classify each study clip by view. In the second step, study clips were processed by a vision transformer to generate clip-level feature vectors, numerical representations of the key imaging characteristics of each clip. Finally, the feature vectors from clips in the study were combined in an aggregation step to produce exam-level diagnostic predictions for LVEF, RVSF, and TR severity.

The view classification model used the established EfficientNet-V2 large architecture,^33^ chosen as the top performer from among similar convolution-based and vision transformer-based models.^33–36^ This model was trained in a fully supervised manner on 10,741 video clips from 1,432 studies, with each clip (typically 100–300 frames) labeled as 1 of 26 predefined TEE views (or the “other” category) through the data annotation process described previously. At test time, the model produced predictions for an evenly sampled subset of frames within a clip, and these frame-level predictions were averaged to generate a single label for the clip. The most likely prediction was assigned as the first-choice view, and the second-most likely prediction was recorded as the second-choice view label.

The diagnostic prediction task consisted of 2 stages. In the first stage, a spatiotemporal vision transformer^37^ generated clip-level feature vectors. The model was trained in a fully supervised manner using short 32-frame segments sampled from video clips from 6,400 training studies as inputs. Each video clip was randomly cropped to a 64-frame segment and then subsampled to select 32 frames spanning approximately a single cardiac cycle. Because the studies include both preintervention and postintervention imaging, clips were temporally stratified into preintervention and postintervention subsets based on acquisition timestamps (clips acquired in the time between the preintervention and postintervention exams were filtered out). Each clip subset was paired with its corresponding preintervention or postintervention reference labels, ensuring that preintervention clips were trained with preintervention labels, and likewise for postintervention clips and labels. Reference labels for biventricular function and TR severity were extracted from the corresponding TEE reports using the LLM-based method previously described.^32^ The 32-frame segment, together with its corresponding reference labels, was passed into the vision transformer to generate a clip-level feature vector.

In the second stage, because each study consisted of multiple clips depicting different views and time points, the clip-level feature vectors were aggregated to produce exam-level diagnostic predictions. Aggregation was performed using a shallow multilayer perceptron network trained with the same report-derived labels. During aggregation, clip-level feature vectors were grouped by intervention phase, and diagnostic predictions were generated separately for preintervention and postintervention imaging. The feature vectors from the clips were summed and normalized before input to the multilayer perceptron. At test time, the model produced exam-level predictions for preintervention and postintervention LVEF, RVSF, and TR severity.

All images were anonymized and stripped of metadata before training. Model training specifics are further detailed in the eSupplement.

### Statistical Analysis: Interobserver Variability

Interobserver variability was quantified using a reserved subset of 400 clips (derived from 20 studies) annotated independently by 2 board-certified cardiac anesthesiologists for view classification. Agreement between raters was quantified in 2 ways: top-1 agreement, when both raters selected the same top-choice view, and top-2 agreement, when the top-choice view of one rater matched either the first or second choice of the other rater. Percent agreement was reported for both measures.

### Statistical Analysis: View Classification

View classification model performance was evaluated on 945 clips drawn from 8 studies reserved for model testing, labeled exclusively by a board-certified cardiac anesthesiologist. View classification performance was evaluated using accuracy, balanced accuracy (to account for differences in view frequencies), top-2 accuracy, and Cohen κ.^38^ Additionally, micro-, macro-, and weighted-averaged values for the following metrics were computed: precision, recall, F1-score, and one-vs-rest multiclass area under the receiver operating characteristic curve (AUROC). Micro-averaging computed the metrics across all clips, macro-averaging computed metrics per view and averaged them equally, and weighted-averaging adjusted for view prevalence. Per-class receiver-operating characteristic (ROC) curves were also calculated using a one-vs-rest approach. All performance metrics were estimated with 10,000 bootstrap resamples of the test set (n = 945 clips) to calculate mean values and 95% confidence intervals (CIs).

### Statistical Analysis: Diagnostic Quantification

For the biventricular function and TR diagnostic tasks, performance was measured on a separate set of 500 reserved studies in which reference labels were obtained by manual parsing of intraoperative TEE reports for preintervention and postintervention LVEF, RVSF, and TR severity values; no LLM extraction was used for these 500 testing reports. Preintervention study predictions were evaluated against preintervention reference labels, and postintervention study predictions were evaluated against postintervention reference labels. These exam-level predictions were assessed for both binary classification and continuous regression tasks.

For LVEF, binary cutoff values of 55% and 30% were selected to reflect thresholds consistent with the lower limit of “normal” LVEF (55%) and the distinction between “moderate” and “severe” LV dysfunction (30%). For RVSF and TR severity, binary classification cutoffs were defined at “normal” versus any degree of dysfunction or regurgitation, as well as less than “moderate” versus any degree of dysfunction or regurgitation “moderate” or worse.^33^ The AUROC was calculated for binary classification tasks, with 95% CIs derived from 10,000 bootstrap resamples.

Continuous regression tasks included exam-level LVEF predictions, which were evaluated using Pearson correlation coefficient (r) and mean absolute error. All regression metrics were bootstrapped (10,000 resamples) to provide mean values and 95% CIs. Agreement and bias between predicted and reference LVEF values were further examined using Bland-Altman analysis. All statistical calculations were performed in Python using the scikit-learn library.

## Results

### View Classification Accuracy and Interobserver Comparison

**Fig 3.**
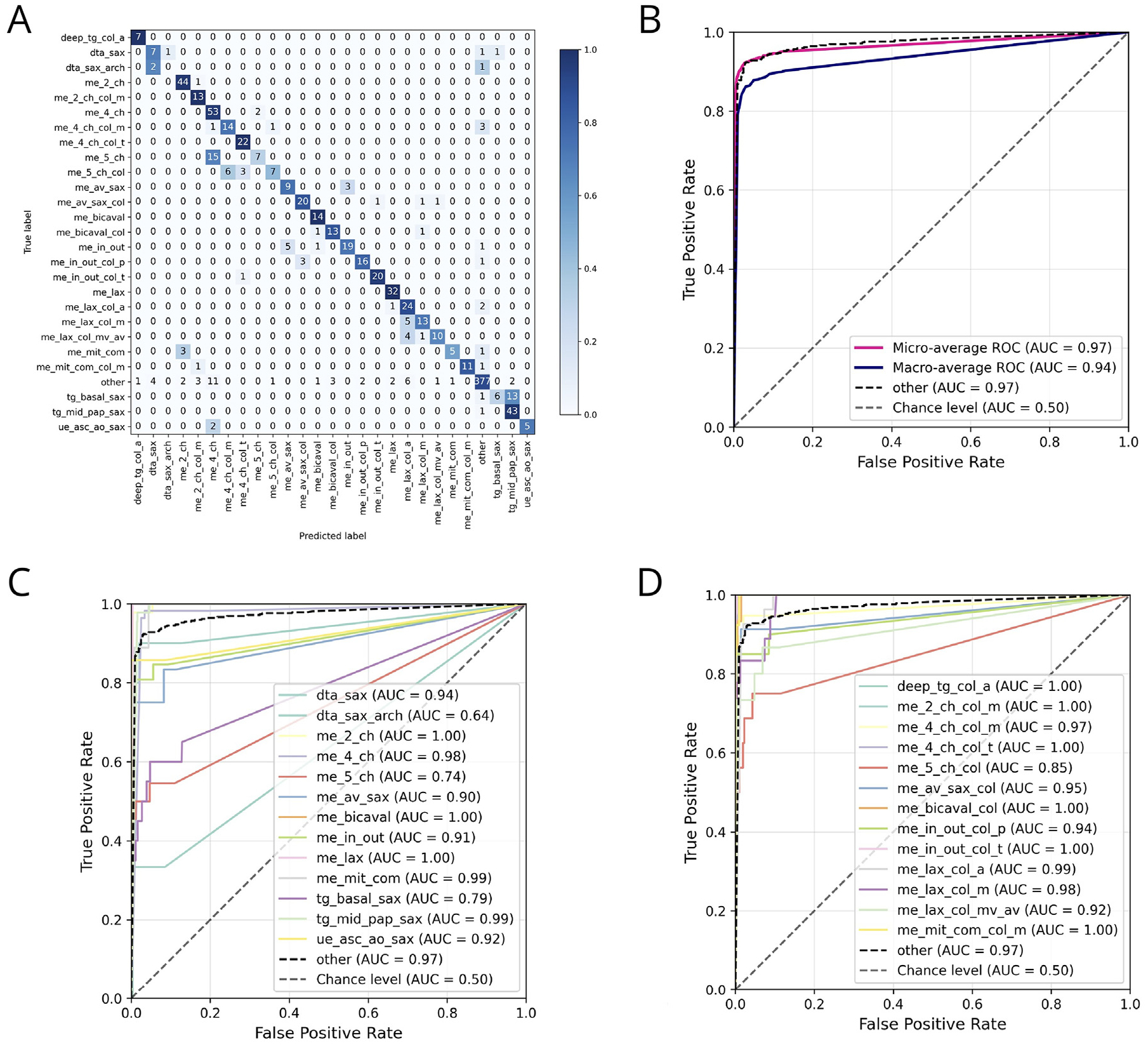
Performance of the view classification model. View label abbreviation labels given in Table 2. (A) A correlation heatmap of view prediction performance compared to reference shows model performance on par with that of a trained human expert. (B) Micro- and macro-averaged ROCs. (C) One-vs-rest ROC curves for noncolor Doppler view classification performance. (D) One-vs-rest ROC curves for color Doppler view classification performance.

Our 26-view classification model achieved strong performance across all averaging schemes. View classification performance results were accuracy = 86% (95% CI = 84%, 88%’ - 84%), balanced accuracy = 77% (95% CI = 73%, 81%), top-2 accuracy = 95% (95% CI = 93%, 96%), and Cohen κ = 0.82 (95% CI = 0.79, 0.85). Per-class accuracies and one-vs-rest ROC curves are shown in Fig 3.

Additionally, the micro-averaged results were precision = 86% (95% CI = 84%, 88%), recall = 86% (95% CI = 84%, 88%), F1-score = 86% (95% CI = 84%, 88%), and one-vs-rest multiclass AUROC = 0.97 (95% CI = 0.96, 0.98). Macro- and weighted-averaged values for precision, recall, F1-score, and one-vs-rest multiclass AUROC are reported in the eSupplement eTable 2.

These results compare with a class-weighted interobserver top-1 agreement of 74% and a weighted top-2 agreement of 84%. Detailed agreement distributions across views are shown in eSupplement eFigure 3.

### Diagnostic Prediction Performance

The diagnostic model differentiated between LVEF >55% (*v* ≤55%), considered the threshold between “borderline” and “normal” function, with an AUROC of 0.86 (95% CI = 0.83, 0.88), and between LVEF >30% (*v* ≤30%), considered the threshold between “moderate” and “severe” dysfunction, with an AUROC of 0.95 (95% CI = 0.93, 0.97). Continuous LVEF prediction achieved a Pearson r of 0.79 (95% CI = 0.75, 0.82) with a mean absolute error of 7.8% (95% CI = 7.4%, 8.3%), and a mean difference of 1.8% (eSupplement eFigure 4).

For RVSF, the model differentiated normal (*v* abnormal) RVSF with an AUROC of 0.82 (95% CI = 0.79, 0.85) and differentiated less than moderate RV dysfunction (*v* moderate dysfunction or greater) with an AUROC of 0.92 (95% CI = 0.88, 0.95). For TR, the model differentiated no TR (*v* any) with an AUROC of 0.69 (95% CI = 0.60, 0.78) and differentiated less than moderate TR (*v* moderate TR or greater) with an AUROC of 0.79 (95% CI = 0.75, 0.82).

**Fig 4.**
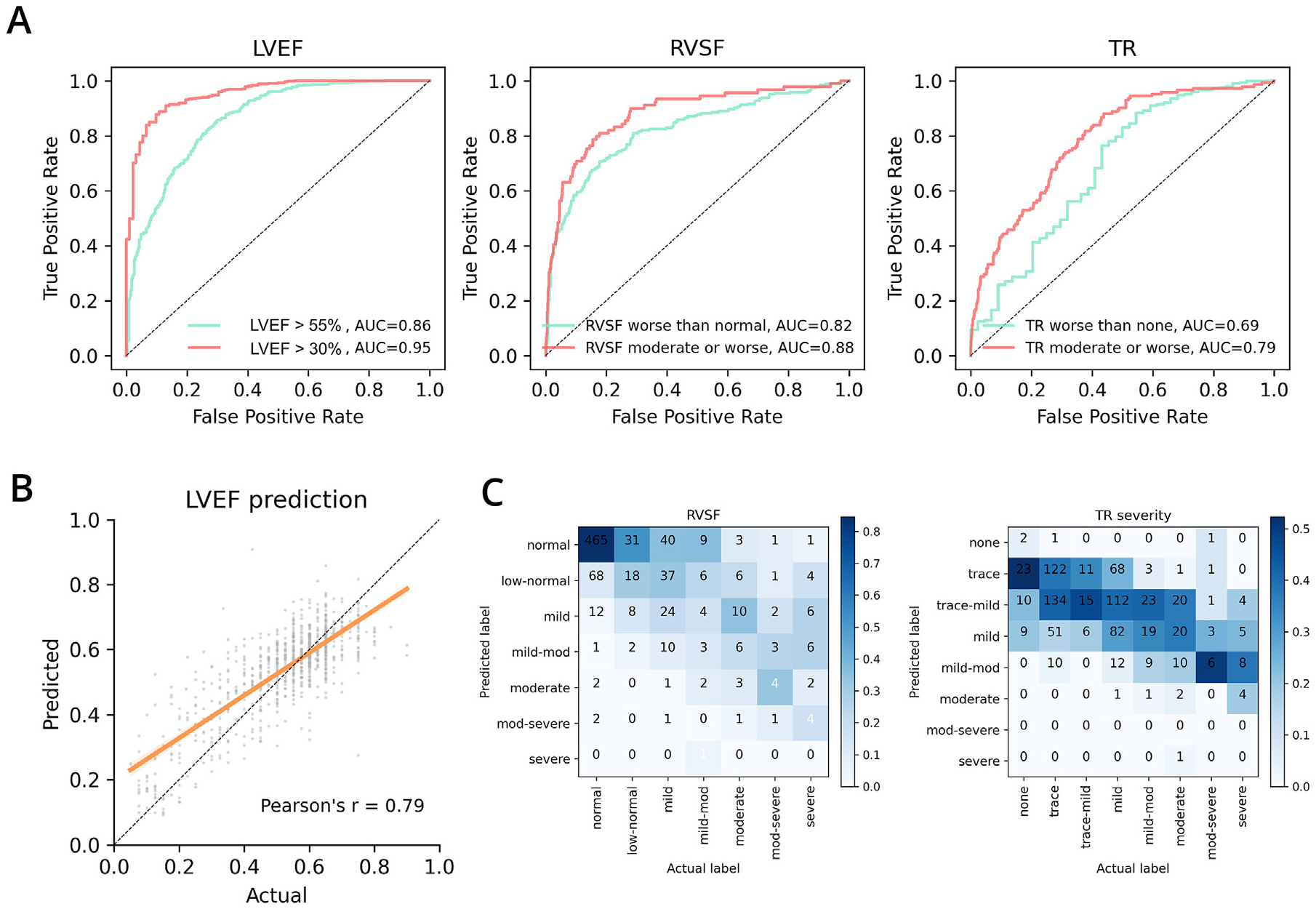
Performance of the diagnostic prediction model. Exam-level diagnostic prediction performance for LVEF, RVSF, and TR severity. (A) ROC curves for binary classification performance predicting LVEF, RVSF, and TR cutoffs. (B) Linear regression of predicted vs reported values for LVEF. (C) Correlation heatmaps for predicted vs reported labels on a standard reporting scale of normal to severe for RV dysfunction and none to severe for TR severity.

ROC curves for all binary classification tasks and regression plots for continuous LVEF prediction are shown in Fig 4. Reported performance reflects both preintervention and postintervention exams, with each exam evaluated against labels from the corresponding intervention phase. ROC curves stratified by preintervention and postintervention exams are shown in eSupplement eFigure 5.

## Discussion

We developed and validated a deep learning-based framework tailored specifically for intraoperative TEE, capable of automated view classification, biventricular function assessment, and TR severity estimation. Our 26-view classification model achieved an accuracy of 86% and a micro-averaged AUROC of 0.97 reflecting strong performance across diverse TEE views. The diagnostic model demonstrated high discriminative ability for identifying patients with LVEF ≤30% (AUROC = 0.95) and for detecting patients with moderate or worse RV systolic dysfunction (AUROC = 0.92). Our diagnostic model demonstrated moderate predictive performance for moderate or worse TR (AUROC = 0.79) and numerical prediction of LVEF (Pearson r = 0.79). These findings represent a key milestone in the development of automated diagnostic tools for complex imaging modalities like TEE.

Our study contributes to the expanding literature on deep learning applications in echocardiography, which has predominantly focused on TTE.^11–17,19,39,40^ One of the earliest deep learning models for TTE view classification was developed by Madani et al. This group achieved a 92% average view classification accuracy across 15 views, using a dataset of 267 studies.^11^ Subsequent efforts have extended this work. For example, Gearhart and colleagues applied deep learning to pediatric TTEs and achieved a 90% average view classification accuracy across 27 views using a dataset of 642 studies.^14^ More recently, Li et al. reported a 98% average view classification accuracy across 6 TTE views in a 2024 study.^16^ While our TEE-specific model achieved a lower micro-averaged view classification accuracy of 86%, direct comparisons between TTE and TEE (particularly intraoperative TEE) view classification are inherently limited due to differences in imaging acquisition, view standardization, and anatomical complexity.

Our work on TEE view classification most closely parallels that of Steffner et al., who developed, validated, and externally tested a deep learning model to classify 8 TEE views using 2,464 video clips.^19^ Although overall view classification accuracy was not reported, their study established an important benchmark for automated TEE interpretation. Our model expands on this foundation by training on a substantially larger dataset comprising more than 10,000 clips from more than 1,400 studies and classifying 26 distinct views. This approach aligned with consensus standards for comprehensive intraoperative TEE.^31^ This granularity preserves clinical specificity, even for anatomically similar views that are frequently confounded by human observers, and better reflects real-world diagnostic complexity. While our results demonstrated higher internal performance (micro-averaged AUROC = 0.97), we did not perform external validation and therefore cannot make direct performance comparisons. Beyond view classification, our framework also incorporated downstream diagnostic prediction of biventricular dysfunction and tricuspid regurgitation severity, representing an extension of prior work toward broader, clinically integrated TEE interpretation.

The incorporation of disease classification into automated TTE deep learning framework has marked a major step forward in echocardiographic AI.^12,13,15,17^ One of the earliest examples was reported by Zhang et al. in 2018, who developed the first method to simultaneously classify 23 TTE views, perform chamber segmentation, and predict cardiac disease.^12^ Their model achieved high AUROC values for predicting hypertrophic cardiomyopathy (0.93), cardiac amyloidosis (0.87), and pulmonary arterial hypertension (0.85).^12^ In 2020, Ghorbani et al. introduced EchoNet, a model that also incorporated downstream tasks, including identification of pacemaker leads (AUROC = 0.89), left atrial enlargement (AUROC = 0.86), and even patient sex (AUROC = 0.88).^13^ However, EchoNet’s performance on continuous variables was more limited, with modest R^2^ values for estimating LVEF (R^2^ = 0.50), age (R^2^ = 0.46), weight (R^2^ = 0.56), or height (R^2^ = 0.33).^13^ To address EF estimation specifically, the same group developed EchoNet-Dynamic, which leveraged LV segmentation to achieve significantly improved EF estimation (R^2^ = 0.77; AUROC = 0.96).^17^

Most recently, Holste et al. published PanEcho, a multitask deep learning system trained on more than 1.2 million TTE videos from more than 32,000 studies.^15^ PanEcho performed 18 automated diagnostic classification tasks (median AUROC = 0.91) and estimated 21 echocardiographic parameters, including robust, automated detection of moderate or worse LV and RV systolic dysfunction (AUROCs up to 0.99 and 0.94, respectively).^15^ It also maintained high diagnostic accuracy across multiple external sites and limited acquisition protocols, including point-of-care settings.^15^

Our TEE-specific model achieves similarly robust performance in biventricular dysfunction assessment and provides moderate diagnostic accuracy for TR severity, representing the first application of such comprehensive AI diagnostic workflows to intraoperative TEE. These comparisons should be interpreted in the context that our model’s diagnostic prediction tasks relied on global qualitative assessments rather than standardized quantitative measurements for ground-truth reference labels.

### Strengths

Our study has several notable strengths. First, it represents the largest TEE-specific dataset used to date for deep learning, encompassing more than 6,400 studies and more than 740,000 video clips. Second, our model predicts 26 distinct TEE views. This far exceeds the 8 views included in the previous TEE study19 and closely mirrors classification schemes common in clinical practice. Third, we deliberately avoided collapsing similar views under pooled labels, resulting in a strategy that increased classification complexity but preserved diagnostic specificity. Fourth, our model extends beyond view classification by incorporating downstream diagnostic tasks, achieving high accuracy for biventricular dysfunction and moderate accuracy for TR severity. These results are on par with recent advances in TTE deep learning, such as EchoNet,^13^ EchoNet-Dynamic,^17^ and PanEcho,^15^ in terms of overall performance metrics, yet applied here for the first time to intraoperative TEE, where real-time guidance can significantly influence surgical and anesthetic decision-making.

### Limitations

Our study has some important limitations. First, although our models were trained and evaluated on a large internal dataset, no external validation was performed, limiting generalizability to other institutions, procedural contexts, and patient populations.

Second, the ground-truth reference labels for diagnostic prediction were extracted from intraoperative TEE reports reflecting expert global qualitative assessments rather than standardized quantitative measurements (e.g., Simpson method, RV fractional area change, or vena contracta width), meaning there was no validation of these assessments. This approach may have introduced interobserver variability and potential bias associated with subjective reporting styles and differing training backgrounds.

Third, all view classification labels were finalized by a single board-certified cardiac anesthesiologist to ensure internal consistency, which may have introduced single-observer bias. A feedback-assisted labeling strategy was also used, where annotators reviewed model-generated view predictions and marked them as correct or incorrect rather than assigning labels de novo, potentially introducing bias.

Fourth, this work focused on view classification and a limited set of diagnostic targets (biventricular systolic function and tricuspid regurgitation), excluding other clinically relevant intraoperative findings such as diastolic function, aortic pathology, prosthetic valve assessment, and shunt quantification. Additionally, diagnostic predictions were aggregated across clips without explicit weighting by image quality or clinical relevance. Individual clips may have reflected physiologic states that differed from the exam-level report-derived labels, introducing noise at the clip level.

Finally, although our models demonstrated strong internal test performance, we did not perform prospective testing or assessment of workflow integration. As with all deep learning applications, model interpretability remains limited. While model confidence and class activation mapping may offer some insight into decision processes, further work is needed to enhance explainability to clinicians and ensure safe, trustworthy integration into perioperative workflows.

## Conclusions

We developed and validated a deep learning framework for intraoperative TEE capable of automated view classification and prediction of biventricular function and tricuspid regurgitation severity. Future work will focus on external validation, model interpretability, and real-time integration into intraoperative workflows. Our findings extend prior efforts in both TTE and TEE imaging by integrating fine-grained classification and diagnostic inference within a unified model trained on a large intraoperative dataset. These results mark an incremental but important step toward automated diagnostic tools for complex imaging modalities such as TEE and may inform future AI-assisted decision support in cardiac surgery.

## Supplementary Material

1

Supplementary material associated with this article can be found in the online version at doi:10.1053/j.jvca.2026.01.036.

## Figures and Tables

**Table 1:** Distribution of Cardiac Surgical Procedures Represented in the Intraoperative TEE Dataset

Procedure Type		Case Count (%)
Isolated AV replacement		423 (8.41%)
AV repair		248 (4.93%)
Isolated MV replacement/repair		671 (13.34%)
AV replacement + MV replacement		56 (1.11%)
AV replacement + CABG		183 (3.64%)
MV replacement + CABG		71 (1.41%)
Isolated CABG		1,252 (24.89%)
Isolated TV procedure		66 (1.31%)
Heart transplant		143 (2.84%)
Lung transplant		127 (2.52%)
VAD	Implanted	160 (3.18%)
	Explanted	36 (0.72%)
ECMO	Venovenous	56 (1.11%)
	Venoarterial	218 (4.33%)
	Venoarterial/venous	4 (0.08%)

NOTE. Surgical categories among the 5,031 intraoperative TEE exams that could be matched to Society of Thoracic Surgeons database.

Abbreviations: AV, aortic valve; CABG, coronary artery bypass graft; ECMO, extracorporeal membrane oxygenation; MV, mitral valve; TV, tricuspid valve; VAD, ventricular assist device (Impella, Heartmate III).

**Table 2:** TEE Views Labels and Abbreviations Used for Model Development and Evaluation

View Abbreviation	Full View Name
deep_tg_col_a	Deep transgastric view with color Doppler (aortic valve)
dta_sax	Descending thoracic aorta short-axis view
dta_sax_arch	Aortic arch short-axis view
me_2_ch	Midesophageal 2-chamber view
me_2_ch_col_m	Midesophageal 2-chamber view with color Doppler (mitral)
me_4_ch	Midesophageal 4-chamber view
me_4_ch_col_m	Midesophageal 4-chamber view with color Doppler (mitral)
me_4_ch_col_t	Midesophageal 4-chamber view with color Doppler (tricuspid)
me_5_ch	Midesophageal 5-chamber view
me_5_ch_col	Midesophageal 5-chamber view with color Doppler
me_av_sax	Midesophageal aortic valve short-axis view
me_av_sax_col	Midesophageal aortic valve short-axis view with color Doppler
me_bicaval	Midesophageal bicaval view
me_bicaval_col	Midesophageal bicaval view with color Doppler
me_in_out	Midesophageal right ventricular inflow–outflow view
me_in_out_col_p	Midesophageal inflow–outflow view with color Doppler (pulmonary)
me_in_out_col_t	Midesophageal inflow–outflow view with color Doppler (tricuspid)
me_lax	Midesophageal long-axis view
me_lax_col_a	Midesophageal long-axis view with color Doppler (aortic)
me_lax_col_m	Midesophageal long-axis view with color Doppler (mitral)
me_lax_col_mv_av	Midesophageal long-axis view with color Doppler (mitral valve and aortic valve)
me_mit_com	Midesophageal mitral commissural view
me_mit_com_col_m	Midesophageal mitral commissural view with color Doppler (mitral)
tg_basal_sax	Transgastric basal short-axis view
tg_mid_pap_sax	Transgastric mid papillary short-axis view
ue_asc_ao_sax	Upper esophageal ascending aorta short-axis view
other	Other (nonstandard or miscellaneous views)

NOTE: The 26 transesophageal echocardiography view labels, along with 1 miscellaneous category, used in this study for model development and evaluation.
