# Supplementary material for: Proof-of-Concept Automated Framework for Intraoperative Transesophageal Echocardiography: View Classification and Biventricular Function Assessment
Source: J Cardiothorac Vasc Anesth. Author manuscript; Available in PMC 2026 Apr 28. (PMC13124233; doi:10.1053/j.jvca.2026.01.036)
Supplement: 1 [file NIHMS2166796-supplement-1.docx]

eSupplement: Proof-of-Concept Automated Framework for Intraoperative Transesophageal Echocardiography: View Classification and Biventricular Function Assessment

**Model Training Details**

All model weights were randomly initialized. All models were optimized using the ADAM optimizer with a dropout rate of 0.2, and a batch size of 64 images. The view classification model and vision transformer used a base learning rate of 5e-5, and the MLP used a base learning rate of 5e-6. To improve model accuracy, random images were augmented through scaling, horizontal flipping, rotation, brightness, and contrast modifications for both the view classification model and the vision transformer during training. For the view classification model, training required approximately 24 hours. For the diagnostic prediction model, training required approximately 48 hours. Training of the MLP for clip prediction aggregation took minutes. All models were trained on a single Nvidia RTX 6000 ADA GPU.

**eTable 1: Distribution of LVEF, RVSF, and TR Represented in the Intraoperative TEE Dataset**

| **Echocardiographic Parameter** ^†^ | | Presurgical | Postsurgical |
| --- | --- | --- | --- |
| **LVSF** | Hyperdynamic (=>75%) | 295 (4.2%) | 561 (7.9%) |
|  | Normal (55 – 74%) | 3580 (50.4%) | 3713 (52.2%) |
|  | Borderline (50 – 54%) | 858 (12.1%) | 656 (9.2%) |
|  | Mild (40 – 49%) | 936 (13.2%) | 830 (11.7%) |
|  | Moderate (31 – 39%) | 325 (4.6%) | 339 (4.8%) |
|  | Severe (<=30%) | 984 (13.9%) | 830 (11.7%) |
|  | Unclassified | 128 (1.8%) | 177 (2.5%) |
| **RVSF** | Normal | 4759 (67.0%) | 4293 (60.4%) |
|  | Borderline / Low Normal | 453 (6.4%) | 568 (8.0%) |
|  | Mild | 801 (11.3%) | 979 (13.8%) |
|  | Mild to Mod. | 170 (2.4%) | 289 (4.1%) |
|  | Mod. | 389 (5.5%) | 433 (6.1%) |
|  | Mod. to Severe | 138 (1.9%) | 139 (2.0%) |
|  | Severe | 204 (2.9%) | 175 (2.5%) |
|  | Unclassified | 192 (2.7%) | 230 (3.2%) |
| **TR** | None | 567 (8.0%) | 552 (7.8%) |
|  | Trivial / Trace | 2576 (36.3%) | 2432 (34.2%) |
|  | Trace to Mild | 222 (3.1%) | 252 (3.6%) |
|  | Mild | 2008 (28.3%) | 2183 (30.7%) |
|  | Mild to Mod. | 365 (5.1%) | 474 (6.7%) |
|  | Mod. | 454 (6.4%) | 434 (6.1%) |
|  | Mod. To Severe | 127 (1.8%) | 83 (1.2%) |
|  | Severe | 252 (3.6%) | 87 (1.2%) |
|  | Unclassified | 535 (7.5%) | 609 (8.6%) |
| ^†^The LVEF, RVSF, and TR categorical distribution for all 6900 imaging studies.  ^‡^Abbreviations: LVEF: left ventricular ejection fraction; RVSF: right ventricular systolic function; TR: tricuspid regurgitation; Mod.: moderate.  ^§^Echocardiographic parameters reflect expert global assessments rather than standardized quantitative measurements (e.g., Simpson’s method, RV fractional area change, or vena contracta width) | | | |
|  |  |  |  |

**eFigure 1: Frequency Distribution of Standard TEE Views in Dataset**


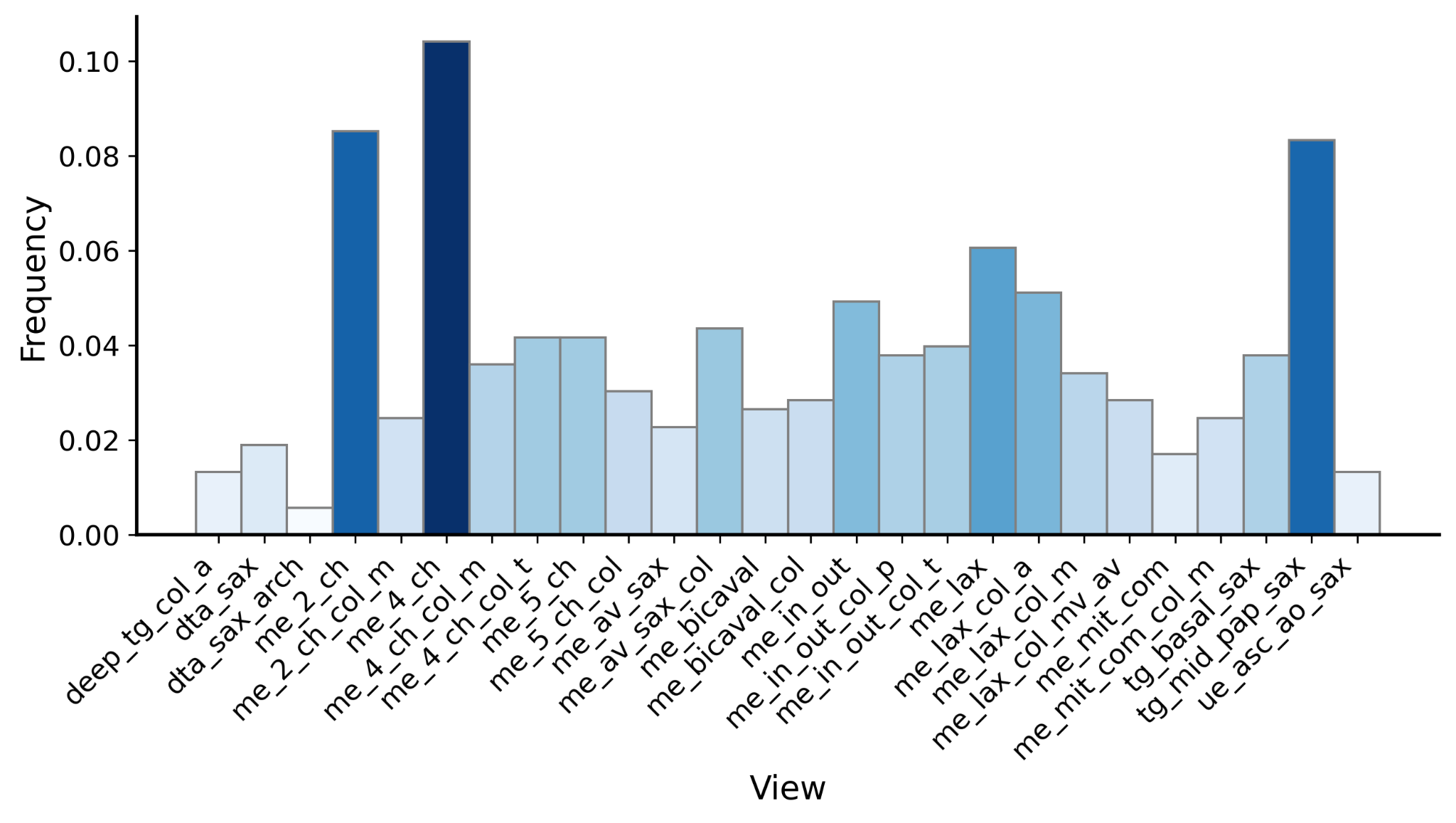


**eFigure 1 Legend**: The frequency of specific views across our dataset was highly unbalanced. In particular, midesophageal 4 chamber, midesophageal 2 chamber, and transgastric mid papillary short-axis, account for an outsize proportion of image clips, while others, such as deep transgastric views and aorta views, are relatively rare.

**eFigure 2: Per-Class Accuracy Versus TEE View Frequency**


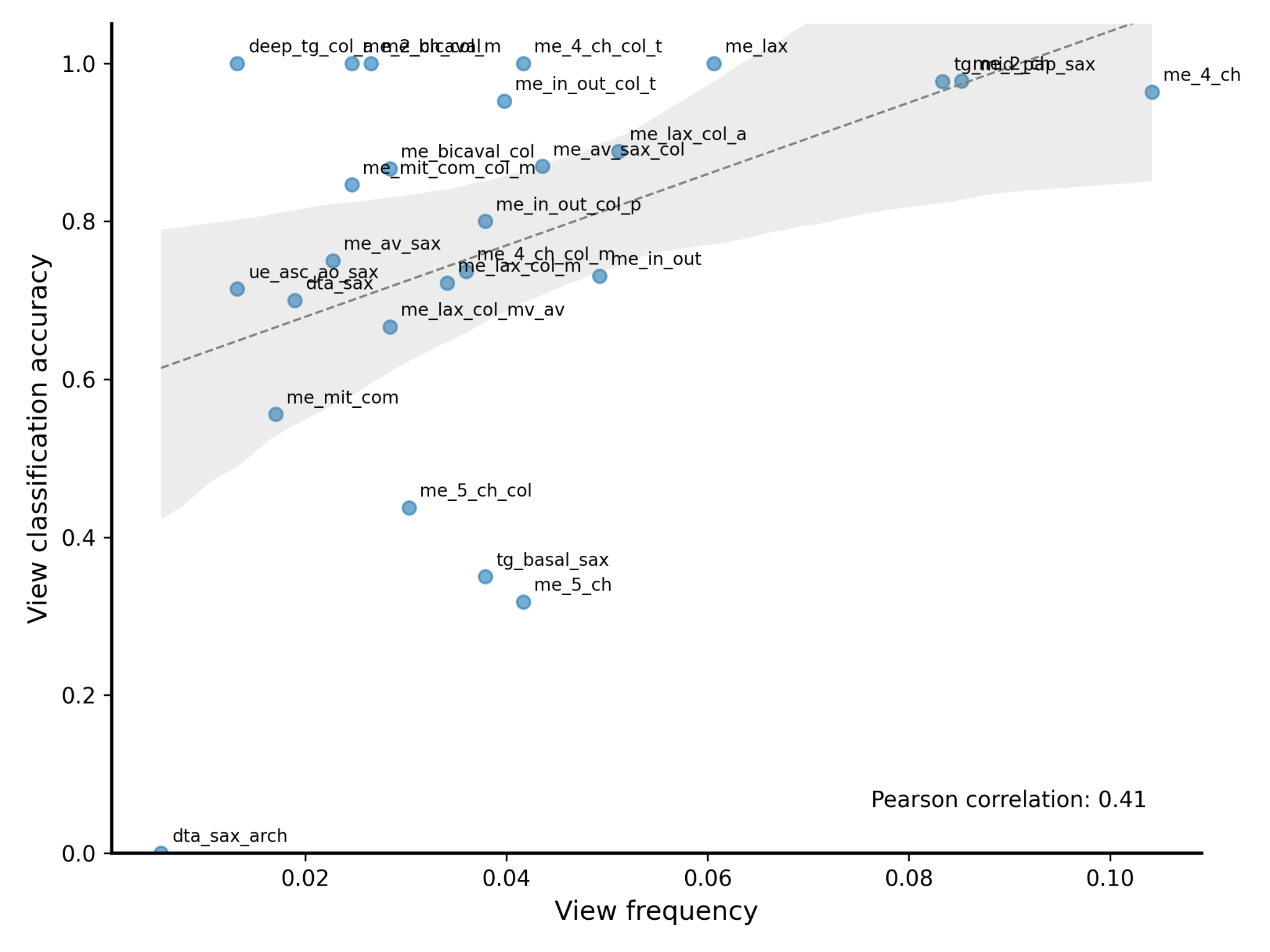


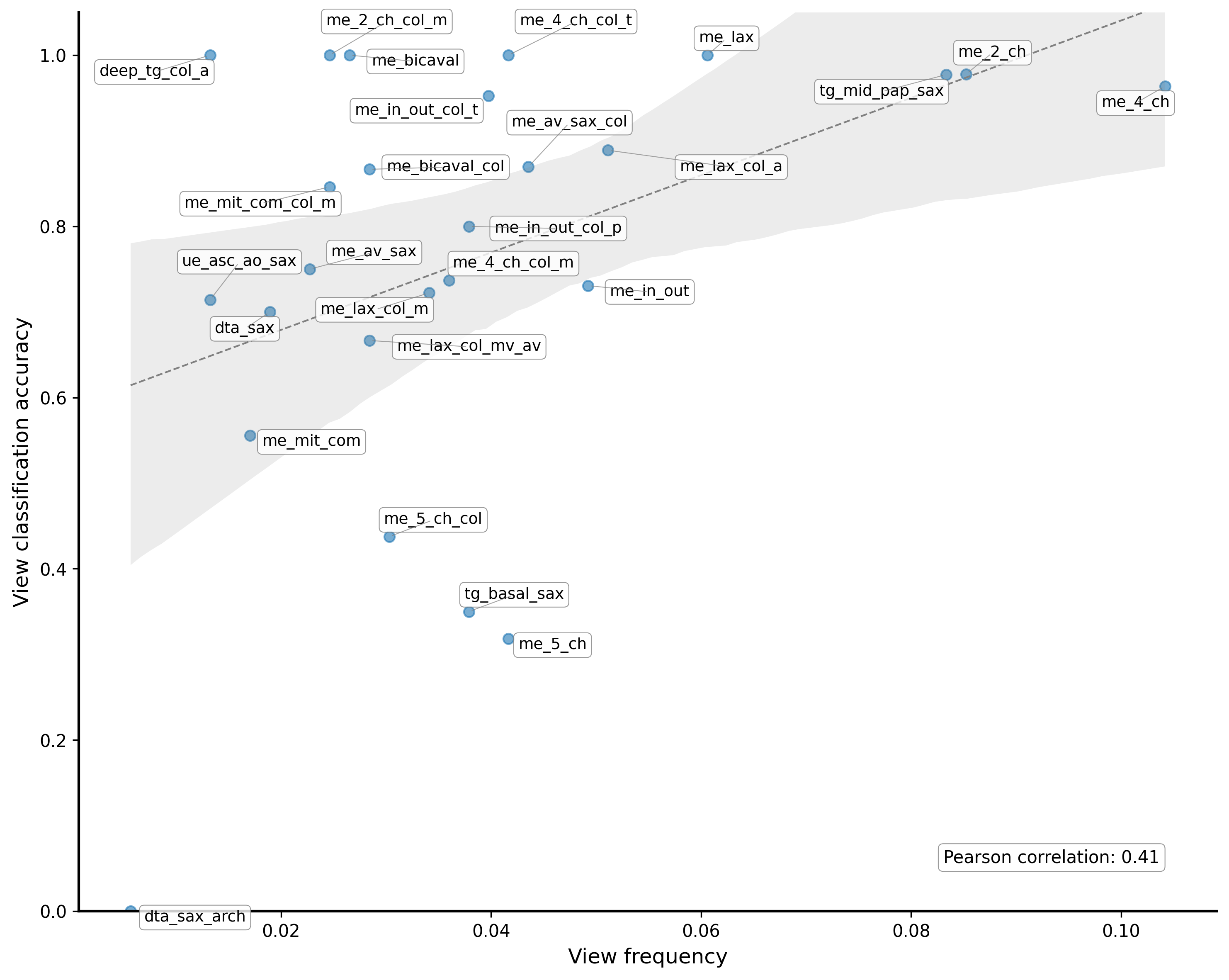


**eFigure 2 Legend**: Per-class accuracy of the view classification model largely correlates with the relative frequencies of each view.

**eFigure 3: Interobserver Agreement in TEE View Annotation**


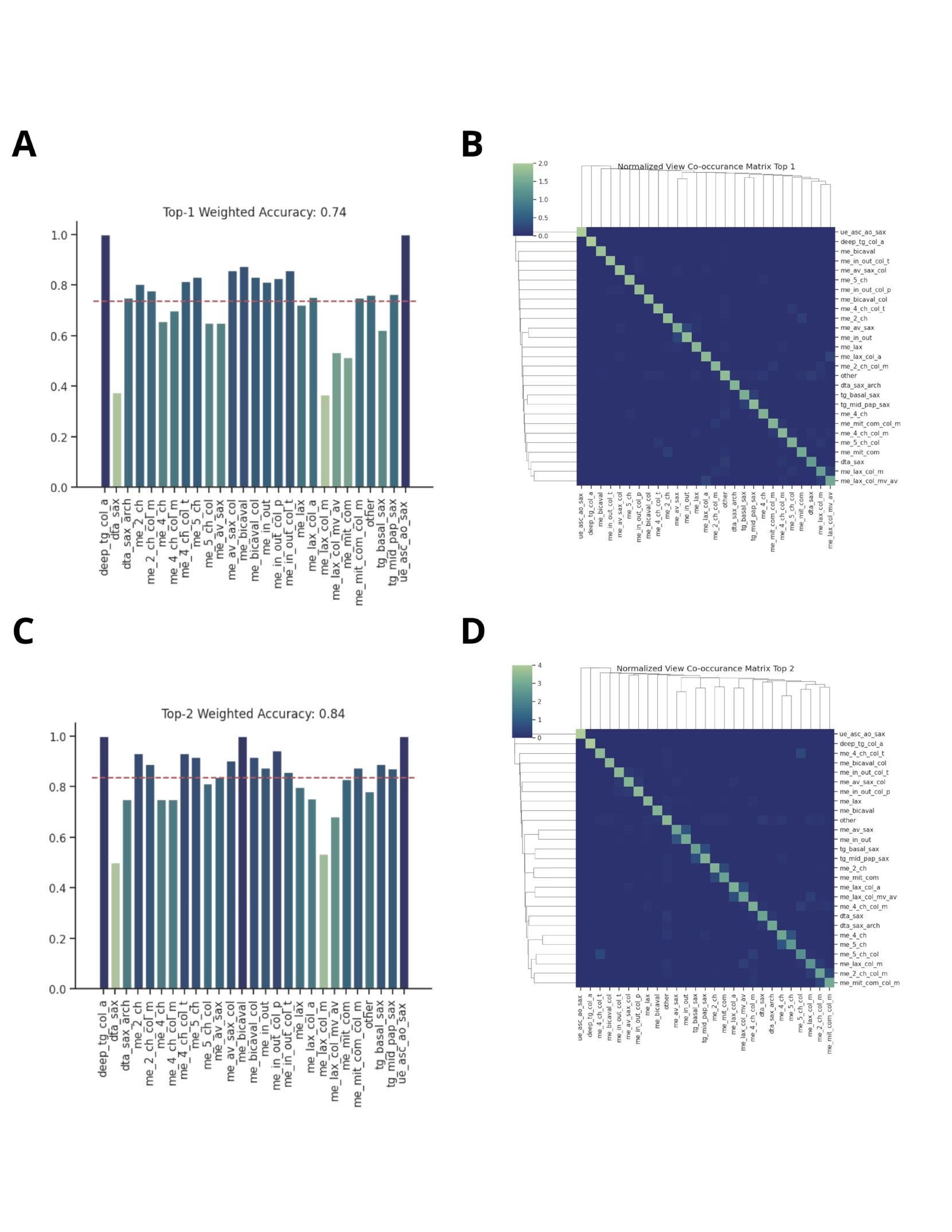


**eFigure 3 Legend**: Interobserver agreement. (A) Interobserver top-1 accuracies across annotated views. (B) Heatmap of view top-1 co-occurrence between annotators. (C) Interobserver top-2 accuracies across annotated views. (D) Heatmap of view top-2 co-occurrence between annotators.


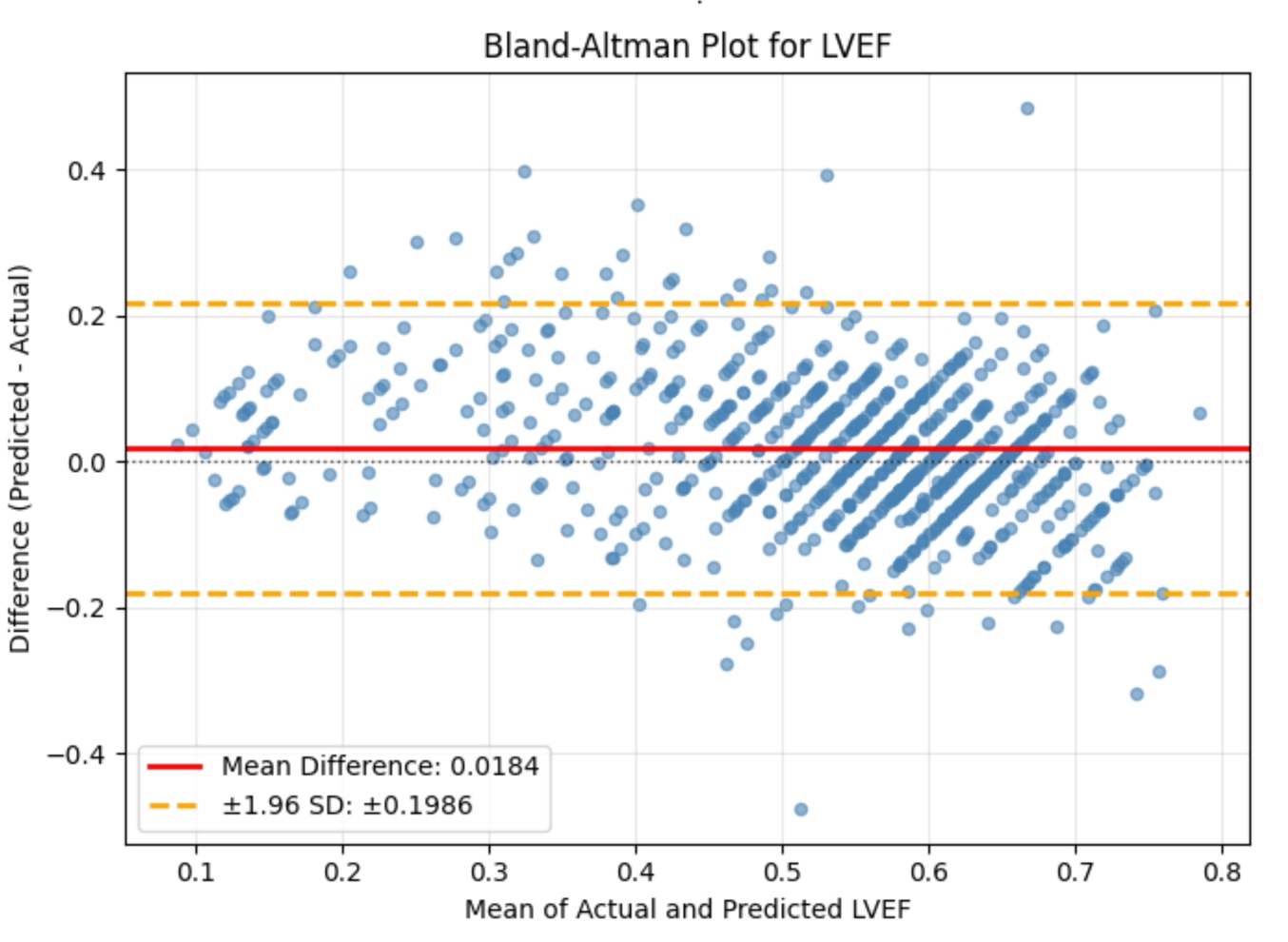
**eFigure 4: Bland-Altman Analysis of LVEF Prediction Accuracy**

**eFigure 4 Legend:** Bland–Altman analysis of agreement between predicted and reference LVEF values.

Abbreviations: LVEF: left ventricular ejection fraction.

**eTable 2: View Classification Performance Across Averaging Schemes**

| **Averaging**  **Scheme**^†^ | **Precision (95% CI)** | **Recall (95% CI)** | **F1-score (95% CI)** | **AUROC (95% CI)** |
| --- | --- | --- | --- | --- |
| **Micro-average** | 86% [84%, 88%] | 86% [84%, 88%] | 86% [84%, 88%] | 0.97 [0.96, 0.98] |
| **Macro-average** | 79% [75%, 82%] | 77% [73%, 80%] | 76% [72%, 79%] | 0.94 [0.92, 0.96] |
| **Weighted-average** | 87% [85%, 90%] | 86% [83%, 88%] | 85% [83%, 88%] | 0.96 [0.95, 0.97] |
| ^†^ View classification performance metrics using micro-, macro-, and weighted-averaging schemes. | | | | |


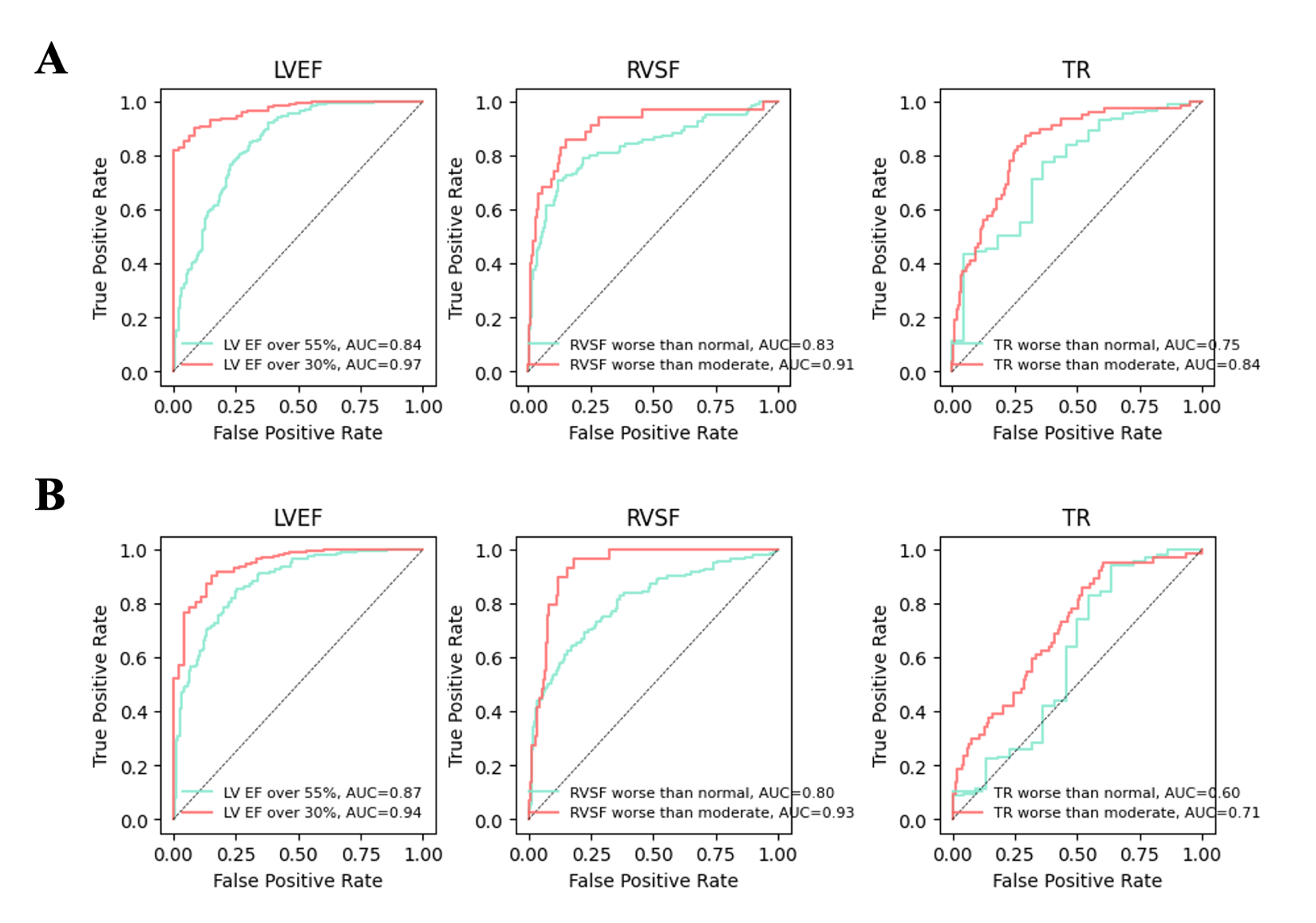


**eFigure 5 Legend:** Exam-level diagnostic performance for prediction of LVEF, RVSF, and TR severity stratified by pre- and post-intervention. ROC curves show binary classification performance predicting LVEF, RVSF, and TR thresholds in the (A) pre-intervention and (B) post-intervention exams.
